# Case Report: Pediatric myeloid/lymphoid neoplasm with eosinophilia and PDGFRA rearrangement: The first case presenting as B-lymphoblastic lymphoma

**DOI:** 10.3389/fped.2022.1059527

**Published:** 2022-12-14

**Authors:** Reem Akiely, Farah Almasri, Nidal Almasri, Amal Abu-Ghosh

**Affiliations:** ^1^ Pediatric Department, King Hussein Cancer Center (KHCC), Amman, Jordan; ^2^ Faculty of Medicine, Jordan University of Science and Technology, Irbid, Jordan; ^3^ Department of Pathology and Laboratory Medicine, King Hussein Cancer Center (KHCC), Amman, Jordan

**Keywords:** myeloid/lymphoid neoplasms, eosinophilia, *PDGFRA*, B-cell lymphoblastic lymphoma (B-LBL), imatinib

## Abstract

According to the latest WHO classification of hematopoietic malignancies, myeloid and lymphoid neoplasms with eosinophilia and gene rearrangements include three specific rare diseases and one provisional entity. Myeloid/lymphoid neoplasms with platelet-derived growth factor receptor alpha (PDGFRA) rearrangements are the most frequent of these disorders and are usually present in adult males with a median age of the late 40s. Patients usually have chronic eosinophilic leukemia but can occasionally manifest as acute myeloid leukemia or extramedullary T- or B-lineage lymphoblastic lymphoma. We report a case of a previously healthy 2-year-old girl who presented with a right supraorbital swelling with no associated lymphadenopathy. Peripheral blood smear evaluation at initial presentation revealed microcytic hypochromic red blood cells and leukocytosis with marked eosinophilia, occasional myelocytes, and occasional blasts. Whole-body CT scans and PET scans revealed hypermetabolic potentially lymphomatous mass in the superior medial aspect of the right orbit in addition to splenomegaly but no evidence of hypermetabolic mediastinal, hilar, abdominal, or pelvic lymph nodes. Bone marrow aspirate and biopsy revealed hypercellular bone marrow with quantitatively decreased erythroid precursors and increased granulocytic precursors with 60% of the cells being eosinophilic cells in different stages of maturation. The diagnosis of myeloid neoplasm with eosinophilia and rearrangement of PDGFRA was made following confirmation by fluorescence *in situ* hybridization (FISH) test for *FIP1L1-PDGFRA* gene fusion. An incisional biopsy of the supraorbital mass revealed B-cell lymphoblastic lymphoma (B-LBL). FISH test for *FIP1L1-PDGFRA* gene fusion was positive in 70% of the cells studied. Thus, the final diagnosis was B-cell lymphoblastic lymphoma arising in the setting of myeloid/lymphoid neoplasm with eosinophilia and PDGFRA rearrangement. The patient was started on imatinib with concomitant therapy for B-LBL per the Children Oncology Group (COG) standard therapy for localized B-LBL and demonstrated a favorable outcome in the 2.5-year follow-up period. To our knowledge, this is the first pediatric case of myeloid/lymphoid neoplasm with PDGFRA rearrangement presenting with synchronous myeloproliferative disease and B-LBL. We present our diagnostic and management approach of this patient and review prior relevant pediatric cases of myeloid/lymphoid neoplasms with PDGFRA rearrangement.

## Introduction

Myeloid/lymphoid neoplasms with eosinophilia (M/LN-Eo) and rearrangements of *PDGFRA*, *PDGFRB*, or *FGFR1*, or with *PCM1-JAK2* genetic variants constitute a rare but well-defined category of hematologic malignancies recognized by the WHO revised classification (1). Despite having myeloproliferative neoplasm (MPN) and eosinophilia as common manifestations in most reported cases, individual patients may display remarkable heterogeneity at initial presentation and clinical course (2). Myeloid/lymphoid neoplasms with PDGFRA rearrangements are the most frequently encountered of these disorders and are usually present in adult males with a median age of the late 40s (1–3). Patients usually have chronic eosinophilic leukemia (CEL) but can also manifest as acute myeloid leukemia (AML), systemic mastocytosis (SM) with hypereosinophilia, or extramedullary T- or B-lineage lymphoblastic lymphoma (T-LBL/B-LBL) (2). Myeloid/lymphoid neoplasms with eosinophilia and gene rearrangements are extremely rare in the pediatric population with only few cases reported in the literature.

Given its exceptional therapeutic benefit demonstrated in previous reports, the tyrosine kinase inhibitor imatinib is currently considered first-line therapy for patients with M/LN-Eo harboring rearrangements of *PDGFRA* (2). However, due to the rarity of these diseases in the pediatric population, and the lack of reports describing imatinib monotherapy in cases presenting with T-LBL/B-LBL, treating physicians are often left with challenging decisions when formulating their management plans. In this report, we present the first pediatric case of myeloid/lymphoid neoplasm with *PDGFRA* rearrangement presenting with synchronous myeloproliferative disease and B-cell lymphoblastic lymphoma (B-LBL). We present our diagnostic and management approach of this patient and review prior relevant pediatric cases of myeloid/lymphoid neoplasms with *PDGFRA* rearrangements.

## Case report

**Figure 1:**
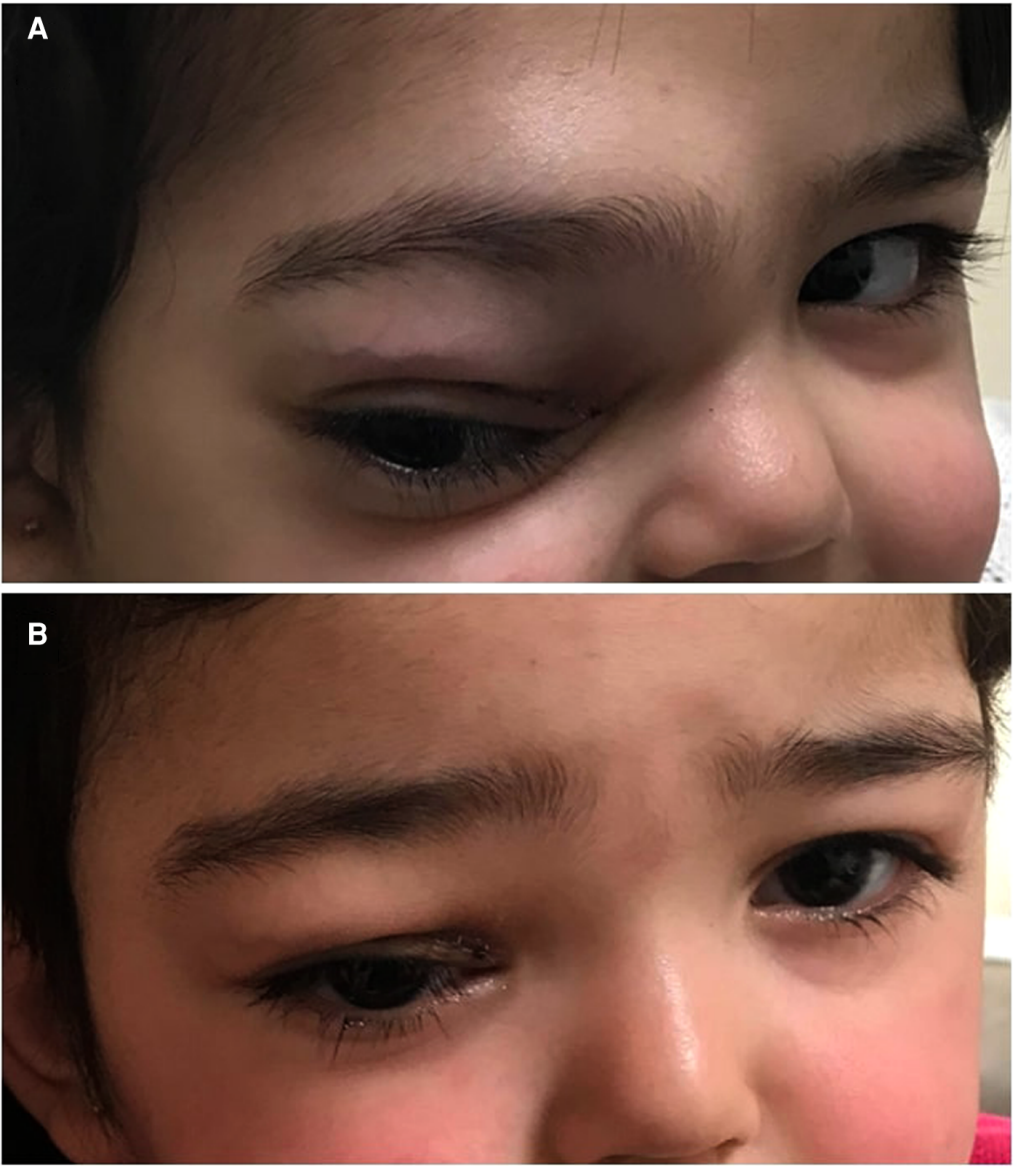
(**A**) Right supraorbital swelling at initial presentation. (**B**) Resolved right supraorbital swelling following 5 months of combined chemotherapy with imatinib.

A previously healthy 2-year-old girl presented with right supraorbital swelling that developed over 1.5 months with no other symptoms at the time of initial presentation. Notable on examination was a right firm supraorbital swelling causing ptosis, with no overlying skin changes (Figure 1A). Examination was also notable for hepatosplenomegaly but not lymphadenopathy. Initial laboratory data were significant for anemia (Hb 8.2 g/dl) and leukocytosis (WBC 19.5 × 10^9^/L) with eosinophilia of 32% and an absolute eosinophilic count of 6.3 × 10^9^/L. Blood smear showed microcytic, hypochromic red blood cells (RBCs) in addition to leukocytosis with marked eosinophilia (32%), occasional myelocytes (2%), and occasional myeloblasts (less than 1%). Some of the eosinophils had three to four nuclear segments and polarized distribution of the cytoplasmic granules.

**Figure 2:**
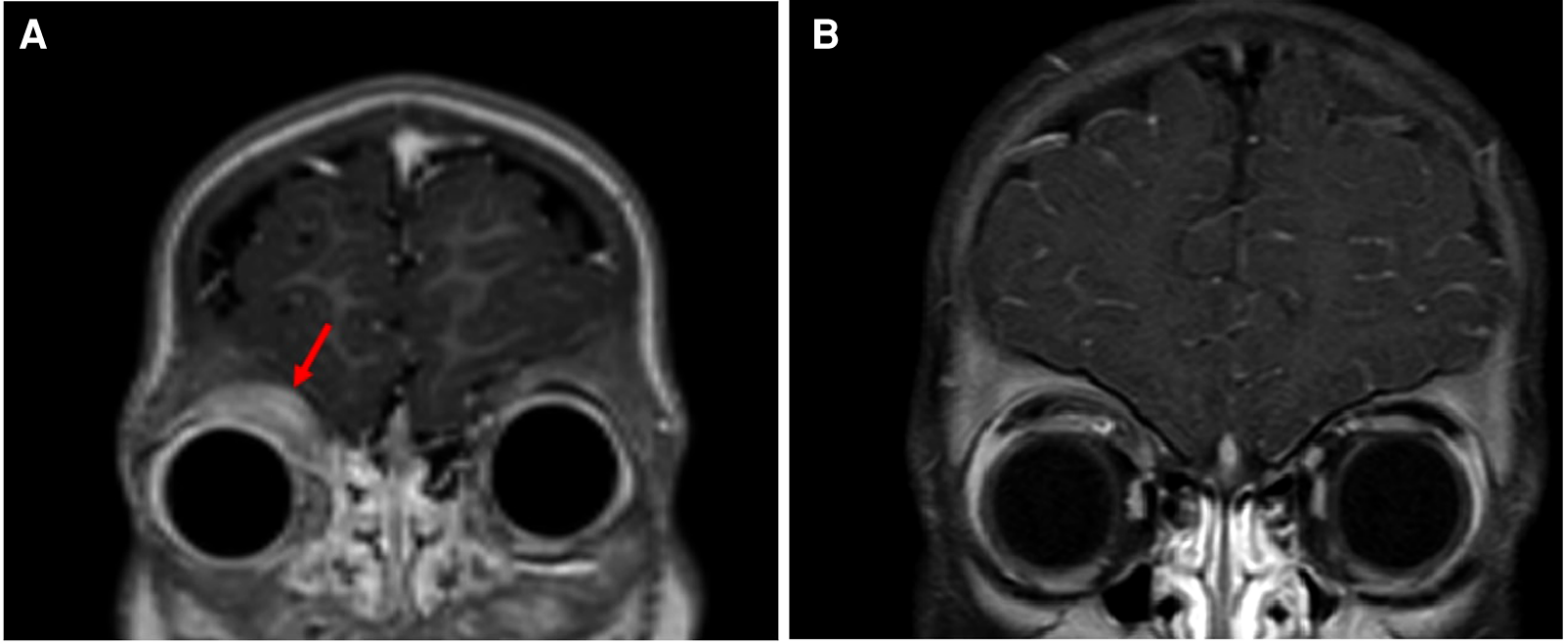
(**A**) Orbital MRI (coronal T1 fat sat postcontrast section) at initial presentation showing mass in the superior medial aspect of the right orbit (arrow). (**B**) Orbital MRI (Coronal T1 fat sat postcontrast section) following 5 months of combined chemotherapy with imatinib demonstrates marked improvement of tumor size.

Whole-body PET/CT scan revealed hypermetabolic potentially lymphomatous mass in the superior medial aspect of the right orbit in addition to splenomegaly, but no evidence of hypermetabolic mediastinal, hilar, abdominal or pelvic lymph nodes. MRI of the orbits revealed a lobulated well-defined nonhomogenous enhancing mass in the superior medial aspect of the right orbit measuring 2.2 cm × 0.6 cm × 2.8 cm. The lesion was mostly involving the anterior aspect of the medial rectus muscle, causing destruction of the medial aspect of the right orbital roof with mild extra-axial intracranial extension but no compression of the brain parenchyma. There was a mild extension of the subcutaneous tissue in the supraorbital space, with compression of the right eye globe but no evidence of invasion of the sclera or retro-ocular extension (Figure 2A).

**Figure 3:**
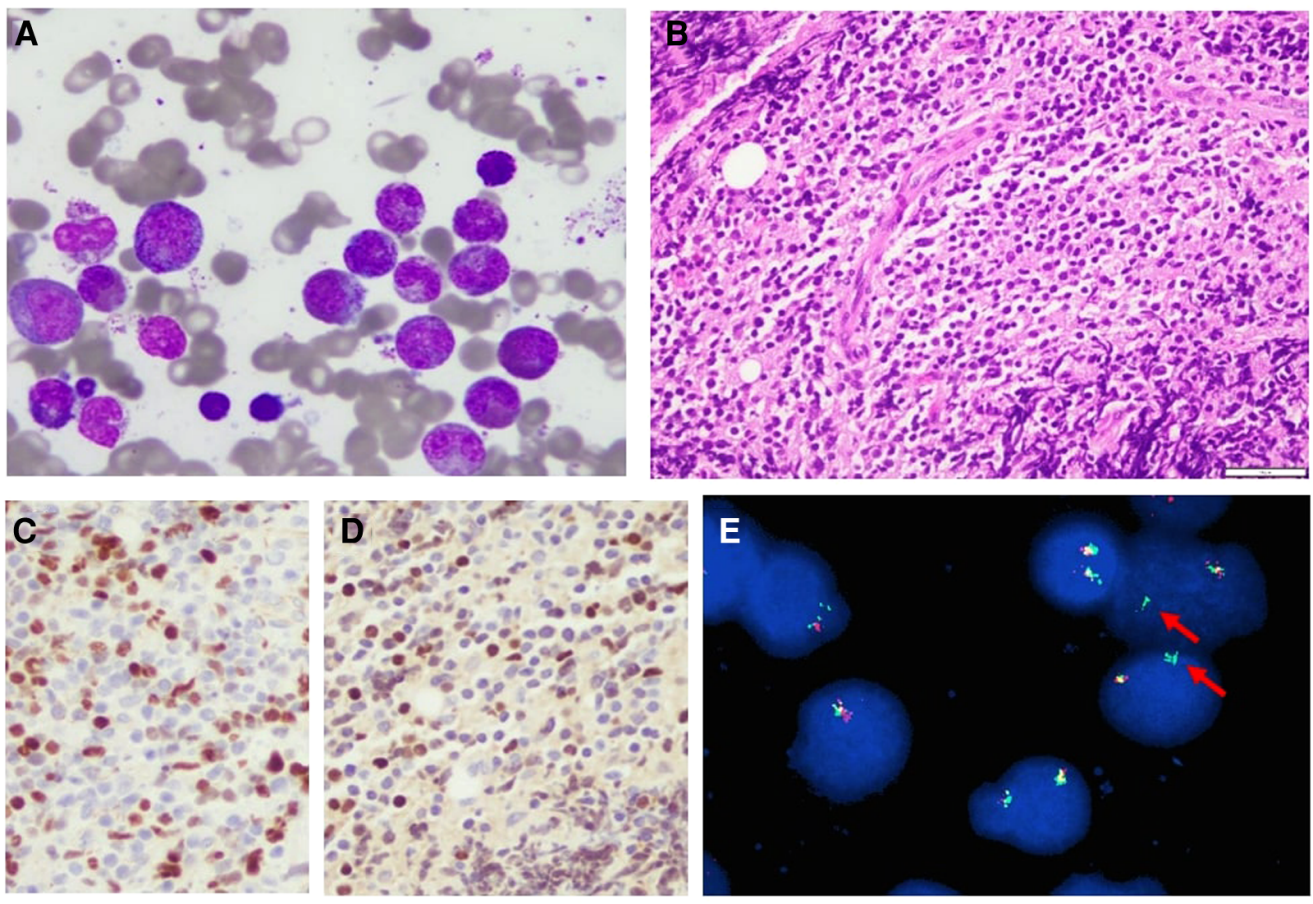
(**A**) Bone marrow aspirate showing mostly eosinophils, Wright–Giemsa stain, 40×. (**B**) Biopsy from the orbital mass demonstrating heavy lymphoid infiltrate composed of small- to medium-sized lymphoblasts, hematoxylin and eosin stain, 40×. (**C,D**) PAX5 and TdT immunostains showing positive brown nuclear stain in the tumor cells, respectively, indicating that these cells are B-cell lymphoblasts 40×. (**E**) Rearranged PDGFRA gene (arrows). Rearrangement results from fusion of FIPL1 and PDGFRA after CHIC2 deletion.

Bone marrow (BM) aspirate and biopsy revealed hypercellular BM with quantitatively decreased erythroid precursors and quantitatively increased granulocytic precursors with 60% of the cells being eosinophilic cells in different stages of maturation. Megakaryocytes were quantitatively normal (Figure 3A). Flow cytometry showed less than 1% myeloblasts. No B-cell lymphoblasts were detected. The diagnosis of myeloid neoplasm with eosinophilia and rearrangement of *PDGFRA* was made following confirmation by fluorescence *in situ* hybridization (FISH) test for *FIP1L1-PDGFRA* gene fusion. Cerebrospinal fluid (CSF) examination showed an acellular specimen with no blast or atypical cells. An incisional biopsy of the supraorbital mass revealed B-cell lymphoblastic lymphoma (B-LBL) (Figure 3B–D). FISH test for *FIP1L1-PDGFRA* gene fusion was positive in 70% of the cells studied (Figure 3E). Thus, the final diagnosis was B-cell lymphoblastic lymphoma arising in the setting of myeloid/lymphoid neoplasm with eosinophilia and *PDGFRA* rearrangement.

Cardiac evaluation was normal. Gastrointestinal evaluation that included upper endoscopy and colonoscopy showed chronic gastric, duodenal, and rectal inflammation but no evidence of eosinophilic infiltration or B-cell lymphoma.

The patient was started on imatinib 340 mg/m^2^/day with concomitant therapy for B-LBL per the Children Oncology Group (COG) standard therapy for localized B-LBL. WBC count and absolute eosinophilic count normalized after 4 days of imatinib. One month after the initiation of therapy, which corresponded to the end of chemotherapy induction phase, the supraorbital swelling decreased grossly to 50% of its original size. The BM evaluation was morphologically normal and the *FIP1L1-PDGFRA* gene fusion by FISH analysis was decreased to 2.5%. Five months following treatment, the supraorbital swelling had completely resolved (Figure 1B). Whole-body PET CT showed almost complete metabolic response, and the brain and orbital MRI showed almost complete resolution of the right orbital mass (Figure 2B). The BM evaluation was also in morphologic remission with negative FISH for *PDFGRA* rearrangement. After 2.5 years of treatment, the multiagent chemotherapy for B-LBL was completed and the patient is now on imatinib monotherapy with close follow-up.

## Discussion

Myeloid/lymphoid neoplasms with eosinophilia and gene rearrangements are extremely rare in the pediatric population with only few cases reported in the literature. To the best of our knowledge, only a few pediatric cases were reported describing myeloid/lymphoid neoplasms harboring *PDGFRA* rearrangements (4–14) (Table 1).

**Table 1:** Summary of reported pediatric cases of myeloid/lymphoid neoplasms with *FIP1L1-PDGFRA* rearrangement.

Reference	Age (years)	Gender	Clinical features at presentation	Initial WBC count (× 10^9^/l)	Initial eosinophilic count (× 10^9^/l)	Disease type	Imatinib dose	Other treatment lines used	Time until achieving hematologic and molecular remission		Follow-up/Long-term outcome
									CHR	CMR	
Rives et al. 2005 (4)	7	Male	Incidental eosinophilia detected on a preoperative blood followed by generalized pruritus and malaise	13.5	6.1	Chronic MPN with eosinophilia/CEL	–	Prednisone	1 month	Not reported	Relapsed after corticosteroids discontinuation but responded to resuming treatment with prednisone, which was maintained for 6 months. Authors intend to treat with imatinib in case of relapse.
Rathe et al. 2010 and 2014 (5, 6)	2	Female	Malaise, fatigue, loss of appetite, and pain	125.0	22.5	Chronic MPN with eosinophilia/CEL	300 mg/m^2^ daily	—	2 weeks	3 months	Continued imatinib for 5 years. Five months after discontinuing therapy, the patient relapsed and imatinib was restarted. CHR and CMR were reached at 4 weeks.
Rapanotti et al, 2010 (7)	16	Male	Lymphadenopathy, splenomegaly, restrictive cardiomyopathy	13.5	5.3	Chronic MPN with eosinophilia/CEL	400 mg daily	—	Achieved with imatinib, timeline not specified		Remained in remission for 36 months
Farruggia et al. 2014 (8)	14	Male	Pallor, weight loss (3 kg in 2 months), and left shoulder pain, lymphadenopathy, hepatosplenomegaly	131.1	49.0	Chronic MPN with eosinophilia/CEL	200 mg daily	Hydroxycarbamide treatment—initiated prior to identifying gene rearrangement—did not control the disease	40 days	8 months	Remained in remission for 12 months
Zeng et al. 2015 (9)	9	Female	Malnutrition, fever, cough, diarrhea, lymphadenopathy, hepatosplenomegaly, progressive skin rash starting at 1 month of age, bilateral keratomalacia with corneal erosions at 3 years, stroke at 7 years, cardiac dysfunction, pneumatosis effusion along within the intestine tract	39.7	28.9	Chronic MPN with eosinophilia/CEL	100 mg daily	Corticosteroid treatment—initiated prior to identifying gene rearrangement—did not control the disease. It was gradually tapered and withdrawn	6 weeks	Not reported	Improved end organs functions at 6 weeks
Oberley et al. 2017 (10)	13	Male	Enlarged right supraclavicular lymph node	1.4	0.0	**T-cell lymphoblastic leukemia/lymphoma**	400 mg daily (added on relapse)	Received standard chemotherapy for T-LBL but had a refractory disease. Imatinib was added after PDGFRA rearrangement was detected. The patient did not respond and died of disease progression at day +217 from diagnosis			
Srinivasan et al. 2019 (11)	15	Male	Migrating joint pain, and leukocytosis, splenomegaly	49.2	16.7	Chronic MPN with eosinophilia/CEL	100 mg daily switched to maintenance dose of 200 mg weekly at around 18 months	Initially started on hydroxyurea prior to identifying gene rearrangement	1 month	3 months	Remained in remission for 42 months since diagnosis and doing well on maintenance imatinib
Bota et al. 2019 (12)	5	Female	Constitutional symptoms, multifocal bone pain, headache, gastrointestinal complaints, hepatosplenomegaly, lymphadenopathy cardiomyopathy	59.7	29.4	Chronic MPN with eosinophilia/CEL	100 mg daily (∼133 mg/m^2^/day) Switched to 100 mg every other day (∼54 mg/m^2^/day) at 9 months	Concomitant methylprednisolone (1 mg/kg/day for 14 days)	3 days	9 months	Remained in remission for 16 months. Cardiomyopathy completely resolved
Jain et al. 2020 (13)	6	Male	Fever, fatigue, massive splenomegaly	18.5	6.1	Chronic MPN, blast phase (23% BM blasts)	100 mg daily (∼100 mg/m2/day)	—	6 weeks	19 weeks	Remained in remission for 2 years since diagnosis
Voeller et al. 2020 (14)	13	Female	Iron deficiency, anorexia, anxiety, frequent panic attacks and exertional shortness of breath, severe restrictive cardiomyopathy with pulmonary hypertension	Not reported—	4–6	Chronic MPN with eosinophilia/CEL	100 mg daily	Steroids	1 month	3 months	Remained in remission after nearly a year on continued monotherapy with imatinib. Remained asymptomatic from a cardiovascular standpoint, but continued to have pulmonary hypertension and severe diastolic dysfunction
Current case	2	Female	Right supraorbital swelling, hepatosplenomegaly	19.5 6.3	6.3	**B-cell lymphoblastic lymphoma**	∼340 mg/m^2^/day	Concomitant therapy for B-LBL per the COG standard therapy protocol	4 days	5 months	Remained in remission for 2.5 years since diagnosis

WBC, white blood cell; CHR, complete hematologic remission; CMR, complete molecular remission; MPN, myeloproliferative neoplasm; CEL, chronic eosinophilic leukemia; COG, children oncology group; PDGFRA, platelet-derived growth factor receptor alpha.

Despite the well-known and very strong male predominance in adult cases of hypereosinophilic syndromes (HES), in general, and myeloid/lymphoid neoplasms with *PDGFRA* rearrangements, in particular, pediatric cases demonstrate a less pronounced male predominance (13, 15). In adults, patients with M/LN-Eo with *PDGFRA* rearrangements usually present as CEL, but can rarely manifest as AML, SM with hypereosinophilia, or extramedullary T-LBL/B-LBL (2). All previously reported pediatric cases manifested as chronic MPN with eosinophilia/CEL with only one case presenting as T-lymphoblastic lymphoma (T-LBL) without eosinophilia (10). To the best of our knowledge, we report the first pediatric case of myeloid/lymphoid neoplasm with *PDGFRA* rearrangement presenting with synchronous myeloproliferative disease and B-lymphoblastic lymphoma (B-LBL).

The product of the *FIP1L1-PDGFRA* fusion gene is a constitutively activated tyrosine kinase, thus, making the tyrosine kinase inhibitor imatinib a well-known agent for targeting conditions with this specific genetic abnormality (16). Imatinib is currently considered the first-line therapy for patients with M/LN-Eo harboring rearrangements of *PDGFRA* (2). In 2005, Rives et al. reported the first child with HES/CEL harboring *FIP1L1-PDGFRA* rearrangement. However, this genetic abnormality was not identified at initial presentation. The patient had received corticosteroids for the diagnosis of primary hypereosinophilic syndrome and achieved complete hematologic response in 1 month. The patient relapsed after discontinuation of the corticosteroids but responded to resuming same treatment. After the *FIP1L1-PDGFRA* fusion gene was identified, the diagnosis was modified to *FIP1L1-PDGFRA*-positive CEL and the authors declared the intent of initiation of imatinib if their patient exhibits a second relapse (4). All subsequent reported pediatric cases included imatinib in their treatment plan (5, 7–14). Favorable outcomes were reported in all cases except the case presenting as T-LBL that was reported by Oberley et al. in 2017 (10). Their 13-year-old male patient had chemotherapy-resistant T-LBL, and once the *FIP1L1-PDGFRA* rearrangement was detected, imatinib was added. However, the patient did not respond and eventually died of his disease (10). Our patient was treated with both imatinib and standard chemotherapy for the B-LBL due to the lack of reports describing imatinib monotherapy in cases presenting with T-LBL/B-LBL in all age groups, in addition to the serious initial presentation of B-LBL that posed an imminent threat to the patient's vision and central nervous system as evident by the aforementioned imaging findings. Five months following treatment, our patient achieved complete clinical, hematologic, and molecular remission. After 2.5 years of treatment, multiagent chemotherapy for B-LBL was completed and the patient is now on imatinib monotherapy and close follow-up.

Discontinuation of imatinib has been associated with relapse in *FIP1L1/PDGFRA*-positive chronic eosinophilic leukemia in both adult and pediatric cases (6, 17, 18). Although few authors reported maintained remission after decreasing imatinib maintenance dose, there is a lack of consensus regarding the optimal maintenance duration (11, 12). It is advised that patients remain on regular follow-up by molecular monitoring to dictate optimal duration of continuation therapy. Moreover, it is generally presumed that patients may require lifelong therapy (6, 13).

## Conclusion

Myeloid/lymphoid neoplasms with eosinophilia and gene rearrangements are extremely rare in the pediatric population with only few cases reported in the literature. In this report, we presented the first pediatric case of myeloid/lymphoid neoplasm with *PDGFRA* rearrangement presenting with synchronous myeloproliferative disease and B-LBL. Our patient demonstrated a favorable outcome after initiating combined chemotherapy with imatinib.

## Data Availability

The original contributions presented in the study are included in the article/Supplementary Material, further inquiries can be directed to the corresponding author.
